# Postauricular versus systemic use of steroids for sudden hearing loss: A systematic review and meta-analysis of randomized controlled trials

**DOI:** 10.1097/MD.0000000000034494

**Published:** 2023-08-11

**Authors:** H.S. Deng, Y.W. Hou, J.N. Zhang, T. Yang

**Affiliations:** a Department of Otolaryngology Head and Neck Surgery, Hanzhong People’s Hospital, Hanzhong, Shaanxi, China; b Chongqing Medical University, Chongqing, China; c Chongqing Institute of Green and Intelligent Technology, Chinese Academy of Science, Chongqing, China; d Chongqing School, University of Chinese Academy of Sciences, Chongqing, China; e Chongqing People Hospital, Chongqing, China.

**Keywords:** meta-analysis, postauricular injection, sudden sensorineural hearing loss, systematic review, systemic therapy

## Abstract

Sudden sensorineural hearing loss (SSNHL) can be debilitating and is one of the most common otological diseases. Steroids play an important role in its treatment. There are many ways to administer steroids, and the efficacy and safety of different administration routes remain unclear. This meta-analysis aimed to investigate the effect and safety of different types of steroid delivery administration for the treatment of SSNHL.

**Methods and analysis:**

We searched the Weipu, Wanfang, Chinese Biomedical Literature, National Knowledge Infrastructure, Web of Science, Embase and PubMed databases for randomized controlled trials (RCTs) on glucocorticoid treatments for SSNHL to compare the efficacy of postauricular injection and systemic steroid administration. Review Manager 5.4 software was used for data synthesis, which included the recovery rate (RR) of reported hearing improvement and change level in pure-tone audiometry (PTA). Subgroup analyses were performed based on different drugs, basic treatment, initial PTA, drug administration methods, onset time, and treatment course. Stata 15.1 software was used for analyses of publication bias and sensitivity.

**Results:**

Our meta-analysis included 38 studies involving 3609 patients with SSNHL. In all included studies, the risk difference (RD) using reported improvement as an outcome measure was 0.12 for postauricular injection administration compared with systemic therapy (95% confidence interval [CI] = 0.008, 0.16, *P* < .00001, I^2^ = 59%). When examining PTA changes as an outcome measure (19 studies), the mean difference was 6.06 (95% CI = 3.96, 8.16, *P* < .00001, I^2^ = 70%). The RD for hearing improvement was compared among different factors, and the results showed that postauricular injection is superior to systemic steroid administration.

**Conclusion:**

Postauricular injection may be safer and more effective treatment than systemic therapy as a treatment for SSNHL.

## 1. Introduction

In 1944, Kleyn first described and defined sudden sensorineural hearing loss (SSNHL) as hearing loss of at least 30 dB HL at 3 or more consecutive frequencies on a pure-tone audiogram in 3 days or fewer.^[1]^ If it cannot be treated in a timely manner, SSNHL can lead to persistent and disabling hearing loss, which significantly compromises the quality of life of patients. In 2021, it was reported by the World Health Organization (WHO) that over 5% of the world population, or 430 million people, required rehabilitation to address their disabling hearing loss (432 million adults and 34 million children). Moreover, it is estimated that by 2050, over 700 million people or 1 in every ten people will have disabling hearing loss.^[2]^ Therefore, studies relating to SSNHL permit no delay.

In 1980, the efficacy of corticosteroid therapy was first confirmed by Wilson.^[3]^ Since then, steroids have been consistently tested in all kinds of clinical and basic trials. Steroids, which serve as the first recommended drug in SSNHL guidelines worldwide, have been widely used for many years. However, their therapeutic efficacy can be greatly affected by the drug administration route, dosage, administration duration time, frequency of application and so on.

**Figure 1.**
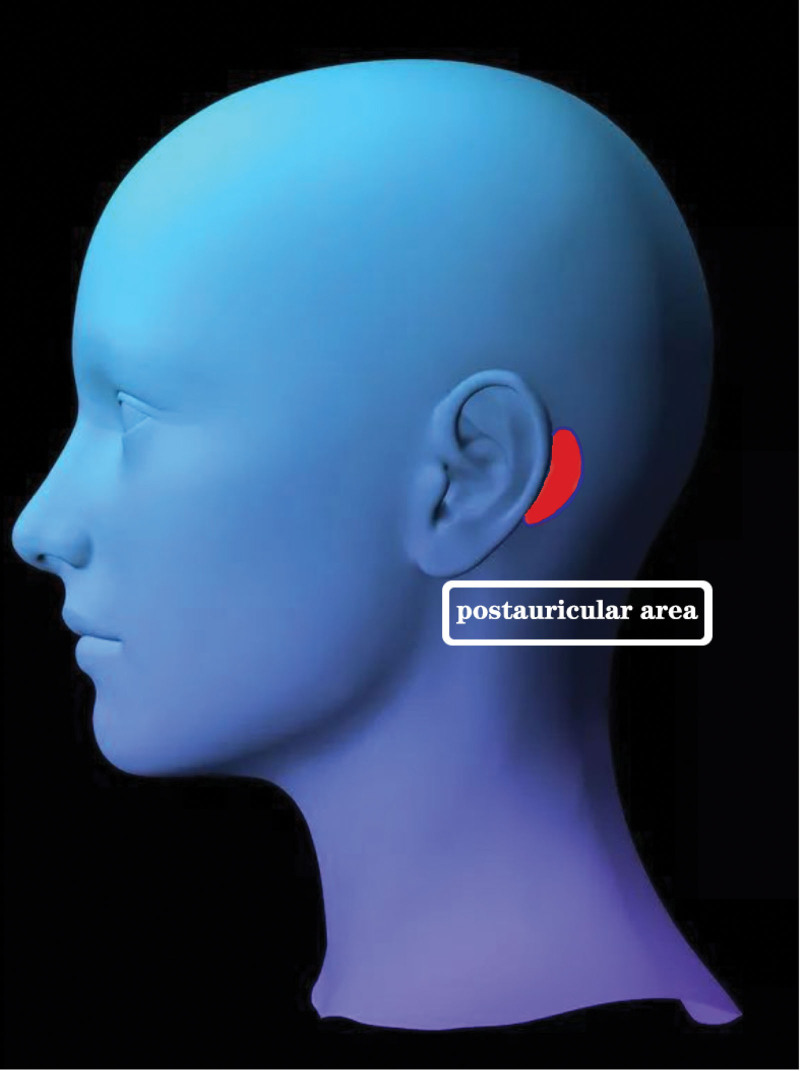
Schematic of postauricular injection.

In terms of the best drug delivery administration, there has been no agreement to date. Currently, there are 2 main methods for steroid administration in SSNHL therapy: systemic steroid therapy and local steroid therapy.^[4]^ The systemic drug delivery method, which is the traditional method of steroid administration for SSNHL, is commonly used and accepted in the clinic. However, the drug delivery efficacy of this method is not ideal and may lead to some side effects, including increased blood pressure, hyperglycemia, electrolyte disturbances, and infections. Therefore, it is more important to identify some local drug delivery methods that can increase targeted drug delivery efficacy and reduce the side effects of steroid application. At present, there are many local drug delivery methods, such as intratympanic steroid therapy, postauricular drug injection (Fig. 1), and intracochlear administration.

The efficacy and safety of postauricular administration have been proven in some clinical trials, and this approach is also more convenient. We aimed to determine whether postauricular administration can be used as an ideal and widely applied therapy for SSNHL. The purpose of this meta-analysis was to evaluate the efficacy and safety of postauricular drug injection for SSNHL treatment by examining published studies in the Chinese and English literature. Postauricular steroid administration may be a more promising method of SSNHL therapy than systemic steroid treatment.

## 2. Materials and methods

### 2.1. Literature retrieval

**Figure 2.**
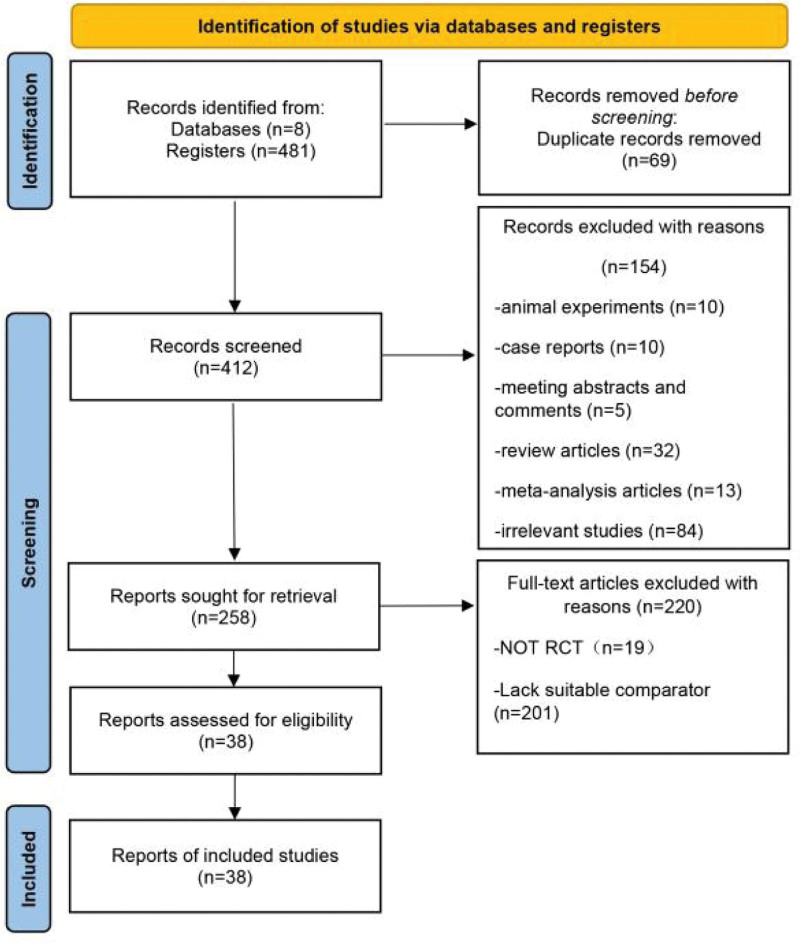
PRISMA flowchart of the review search. PRISMA = Preferred Reporting Items for Systematic Review and Meta-analysis Protocols.

This study was designed based on the Preferred Reporting Items for Systematic Review and Meta-analysis Protocols statement, and the protocol was reviewed and registered in PROSPERO (ID: CRD42022343333). Two investigators (YT and DHS) independently searched the Weipu, Wanfang, Chinese Biomedical Literature, National Knowledge Infrastructure, Web of Science, Embase and PubMed databases up to May 1, 2022. The search was limited to English and Chinese articles. Free search terms and Medical Subject Headings terms were both used in the literature search. The following search terms were used: “sudden hearing loss,” “sudden deafness,” “sudden sensorineural hearing loss,” “idiopathic sensorineural hearing loss,” “idiopathic deafness,” “postauricular,” “postauricular injection administration,” “opisthotic injection,” “systemic,” “systemic administration,” “intravenous injection,” “intravenous drip,” “hormone, hormones,” “hormone therapy, phytohormone,” “glucocorticoid,” “glucocorticoid hormone,” and “glucocorticoids.” The flow chart of the study selection process is shown in Figure 2.

### 2.2. Study selection

Two investigators (YT and HYW) independently screened the identified abstracts and selected studies for full review. Full-text review, which included intensive reading of the appropriate articles, was performed by the same investigators. All disagreements were resolved by group consensus.

### 2.3. Inclusion and exclusion criteria

#### 2.3.1. Research categories.

All the included studies were randomized controlled trials (RCTs) published in English or Chinese, and only human studies were included.

#### 2.3.2. Research subjects.

All patients with SSNHL who had normal behavior and the ability to communicate were included. There were no limitations on race, age, sex or disease course. Patients who had some basic diseases, including hypertension, diabetes and blood diseases, that were well controlled were all included. However, patients diagnosed with stroke, nasopharyngeal carcinoma, acoustic neuroma, otitis media, inner ear malformation, active peptic ulcer, tuberculosis, or a psychological disorder were excluded.

#### 2.3.3. Interventions.

All the included experimental groups were administered steroids via a retroauricular subperiosteal injection in the periosteum of the posterior plain mastoid region or a cribriform area injection. Oral or intravenous steroid therapy was used in the control group.

#### 2.3.4. Outcome assessment criteria.

Pure-tone audiometry (PTA) was performed before and after treatment. The categories of the outcome^[5]^ were as follows: recovery: hearing threshold improved to a normal level after the treatment; obvious effectiveness: an improvement in the hearing threshold of more than 30 dB HL; effectiveness: an improvement in the hearing threshold of 15 to 30 dB HL; and ineffectiveness: an improvement in the hearing threshold of <15 dB HL.

#### 2.3.5. Exclusion criteria.

The exclusion criteria were as follows: articles that were not published in English or Chinese; articles that had no key information, such as the lack of a suitable comparator and the main quantitative outcomes; animal experimental investigations, case reports, meeting abstracts and comments, meta-analyses and review articles; duplicate articles; and studies that were not RCTs.

#### 2.3.6. Data extraction and outcome definitions.

Two investigators (YT and ZJN) independently extracted the data, and any disagreements were resolved by discussion and consensus with the third investigator (DHS). For each selected publication, the following baseline and study characteristics were extracted: sample size, mean age, time to onset, affected ear, initial PTA, country, treatment information (e.g., SSNHL definition, drug condition, mode of administration, duration of treatment, dose), and treatment efficacy (e.g., criteria for hearing recovery, recovery rate (RR), PTA differences and follow-up period).

#### 2.3.7. Risk-of-bias assessment.

The Cochrane Collaboration tool was used to assess the risk of bias in the selected studies using the Cochrane Hand Book 5.4 software.^[6]^ The following aspects were assessed independently by 2 reviewers (YT and DHS): random sequence generation, allocation concealment, blinding of participants and personnel, blinding of outcome assessment, incomplete outcome data, selective reporting, and other bias. Disagreements between the 2 reviewers were resolved through either discussion or adjudication by a third reviewer (ZJN). We judged each study as having a low, unclear, or high risk of bias in each domain. In the review of randomization, studies that described their exact randomization method were scored as having a low risk of bias; however, when the study did not report the exact randomization method but indicated that they had randomized, controlled designs, we scored them as “unclear.” Similar criteria were applied for the scoring of allocation concealment, blinding, incomplete outcome data, and selective reporting.

#### 2.3.8. Statistical methods.

RevMan 15.4 and Stata 15.1 software were used for the data analyses. We pooled the data and used the mean difference with the 95% confidence interval (CI) to assess the continuous variables, for example, PTA changes in dB HL; the risk difference (RD) with the 95% CI was used to assess dichotomous variables, for example, hearing recovery. Heterogeneity was evaluated using the chi-square test.

Heterogeneity between studies was also assessed using the I^2^ value, which indicated the likelihood that the variability between difference effect estimates exceeded expectations. The I^2^ values were categorized as follows using the Nordic Cochrane Centre reference:

I^2^ = 0% to 40%, no important heterogeneity; I^2^ = 30% to 60%, moderate heterogeneity; I^2^ = 50% to 90%, substantial heterogeneity; I^2^ = 75% to 100%, considerable heterogeneity.

An I^2^ statistic ≥ 50% and a Cochrane Q statistic with a value of *P* < .1 indicated the presence of heterogeneity, and in such cases, the random effects model was used. If no considerable heterogeneity among studies was apparent (*P* ≤ .1, I^2^ ≥ 50%), the fixed effects model was used. Funnel plots and Egger linear regression test were used to investigate potential publication bias (n ≥ 10). All statistical analyses were carried out with Review Manager 5.4 (The Cochrane Collaboration).

## 3. Results

### 3.1. Search results and study characteristics

A total of 481 citations were identified from the Weipu, Wanfang, China National Knowledge Infrastructure, Cochrane Library, Web of Science, PubMed and Embase databases. The number of citations decreased to 412 after duplicates were removed. By screening titles and abstracts, we excluded an additional 154 articles for different reasons, including animal experiments, case reports, meeting abstracts and comments, review articles and irrelevant studies. Of the 258 potentially eligible studies, 220 articles were excluded for the following reasons: the study was not an RCT and/or the lack of a suitable comparator. Ultimately, 38 studies^[7–44]^ with 3609 participants were included. All the studies were published in Chinese from 2013 to 2021. The search strategy is shown in Figure 2, and descriptions of the studies are summarized in Table 1. The treatment and outcome details of the selected studies are shown in Tables 2 and 3.

**Table 1:** Characteristics of the eligible studies.

Study (publication yr)	Country	Sample size (n)		Mean age		Time to onset (d)		Affected ear		Sex		With tinnitus (n)		With vertigo (n)		Initial PTA (dB)		With hypertension (n)		Diabetic population (n)	
		T	C	T	C	T	C	T	C	T	C	T	C	T	C	T	C	T	C	T	C
Pan 2013-1^[7]^	China	15	15	17.00~75.00		NA	NA	6 (L)/9 (R)	6 (L)/9 (R)	6 (M)/9 (F)	8 (M)/7 (F)	12	13	3	6	73.5 ± 23.19	72.27 ± 20.08	NA		NA	
Pan 2013-2^[7]^	China	15	15	17.00~75.00		NA	NA	6 (L)/9 (R)	8 (L)/7 (R)	6 (M)/9 (F)	8 (M)/7 (F)	12	13	3	5	73.5 ± 23.19	72.49 ± 21.50	NA		NA	
Chen 2014^[8]^	China	35	37	18~68 (AVG:40.26)	18~63 (AVG:38.14)	1~30 (AVG:6.20)	1~30 (AVG:6.40)	NA		14 (M)/21 (F)	15 (M)/22 (F)	NA		NA		NA		0		0	
Wang 2014^[9]^	China	218	220	44.50 ± 25.20	46.60 ± 23.40	8.50 ± 6.70	10.30 ± 6.70	112 (L)/106 (R)	109 (L)/111 (R)	117 (M)/101 (F)	124 (M)/96 (F)	NA		NA		NA		63	61	NA	
Cao 2015^[10]^	China	27	31	51.70 ± 22.40	49.20 ± 21.60	6.40 ± 4.50	5.80 ± 4.50	13 (L)/14 (R)	18 (L)/13 (R)	15 (M)/12 (F)	17 (M)/14 (F)	NA		NA		75.30 ± 24.60	70.70 ± 23.8	NA		NA	
Dong 2015-1^[11]^	China	31	30	46.16 ± 12.15	47.23 ± 12.76	NA		14 (L)/17 (R)	15 (L)/15 (R)	15 (M)/16 (F)	15 (M)/15 (F)	NA		NA		NA		NA		NA	
Dong 2015-2^[11]^	China	25	22	46.80 ± 11.36	46.77 ± 12.87	NA		11 (L)/14 (R)	9 (L)/13 (R)	11 (M)/14 (F)	13 (M)/9 (F)	NA		NA		NA		NA		NA	
Qu 2015^[12]^	China	62	69	18.00~65.00		Within 14 days		NA		30 (M)/3 2 (F)	36 (M)/33 (F)	29	33	14	13	NA		13	11	9	8
Qin 2015^[13]^	China	30	32	45.87 ± 15.00	47.16 ± 14.92	NA		NA		15 (M)/15 (F)	16 (M)/16 (F)	NA		NA		61.77 ± 19.70	63.72 ± 19.95	0		0	
Wu 2015^[14]^	China	42	38	42.43 ± 15.18		Within 14 days		40 (L)/35 (R)/5 (D)		3 1 (M)/49 (F)		56		23		67.29 ± 22.47	70.52 ± 22.76	38		0	
Cai 2016^[15]^	China	41	44	60.10 ± 5.30	59.40 ± 6.10	NA		NA		19 (M)/22 (F)	21 (M)/23 (F)	NA		NA		67.93 ± 10.43	68.34 ± 11.36	NA		41	44
Fang 2016^[16]^	China	43	44	39.50 ± 8.60	41.30 ± 9.40	7.70 ± 2.40	7.40 ± 1.90	NA		18 (M)/25 (F)	23 (M)/21 (F)	NA		NA		71.90 ± 9.20	69.80 ± 10.70	NA		NA	
Jia 2016^[17]^	China	30	30	50.32 ± 11.68	51.28 ± 10.35	4.21 ± 1.52	4.87 ± 1.98	15 (L)/15 (R)	13 (L)/18 (R)	14 (M)/16 (F)	14 (M)/16 (F)	NA		NA		71.19 ± 20.57	69.83 ± 23.17	20	17	12	11
Li 2016^[18]^	China	39	39	49.69 ± 9.04	47.68 ± 8.65	NA		NA		28 (M)/11 (F)	29 (M)/10 (F)	12		23		NA		NA		NA	
Lu 2016^[19]^	China	35	35	46.02 ± 6.29	46.11 ± 6.35	NA		NA		18 (M)/17 (F)	19 (M)/16 (F)	NA		NA		61.25 ± 8.29	61.33 ± 8.31	NA		NA	
Zhang 2016^[20]^	China	35	43	42.6 ± 18.0	40.23 ± 16.70	17.6 ± 5.30	16.02 ± 7.80	20 (L)/15 (R)	19 (L)/24 (R)	19 (M)/16 (F)	22 (M)/21 (F)	NA		NA		73.90 ± 2.20	78.50 ± 2.85	9		3	
Zhou 2016^[21]^	China	32	32	48.40 ± 0.60	53.40 ± 3.80	7.00 ± 0.20	8.20 ± 1.00	NA		18 (M)/14 (F)	15 (M)/17 (F)	NA		NA		74.20 ± 11.80	70.80 ± 18.40	NA		NA	
Chen 2017^[22]^	China	54	48	41.50 ± 23.40	42.60 ± 20.20	8.70 ± 5.70	10.30 ± 6.70	NA		23 (M)/31 (F)	20 (M)/28 (F)	NA		NA		26.17 ± 6.76	28.03 ± 5.83	11	8	9	4
Wei 2017^[23]^	China	40	40	58.80 ± 7.70	58. 00 ± 7.90	0.50~14.00	1.00~13.00	32 (L)/48 (R)		NA		NA		NA		56.80 ± 8.70	54.20 ± 9.30	0		0	
Zhao 2017^[24]^	China	47	47	50.20 ± 10.70	49.50 ± 11.30	1.59 ± 0.51	1.63 ± 0.56	NA		15 (M)/32 (F)	15 (M)/32 (F)	NA		NA		50.50 ± 13.10	52.30 ± 11.20	NA		NA	
Zhu 2017^[25]^	China	35	31	39.09 ± 11.50	40.32 ± 12.87	6.74 ± 6.67	5.58 ± 5.07	18 (L)/17 (R)	19 (L)/12 (R)	22 (M)/13 (F)	18 (M)/13 (F)	31	29	6	6	75.52 ± 22.22	74.63 ± 20.07	0		0	
Li 2018^[26]^	China	35	35	45.67 ± 5.23	45.28 ± 5.51	10.91 ± 4.02	10.53 ± 3.74	NA		19 (M)/16 (F)	20 (M)/15 (F)	NA		NA		70.63 ± 8.43	70.15 ± 8.61	0		0	
Wang 2018^[27]^	China	40	40	16.64 ± 1.63	16.78 ± 1.55	Within 14 d		19 (L)/21 (R)	20 (L)/20 (R)	20 (M)/20 (F)	21 (M)/19 (F)	NA		NA		NA		0		0	
Zhan 2018^[28]^	China	84	83	54.30 ± 5.80	55.80 ± 4.70	2.00~30.00	3.00~35.00	NA		47 (M)/37 (F)	43 (M)/40 (F)	NA		NA		NA		NA		NA	
Zhang-1 2018^[29]^	China	15	15	46.30 ± 7.00	45.90 ± 7.20	NA		NA		8 (M)/7 (F)	8 (M)/7 (F)	NA		NA		61.28 ± 8.30	61.25 ± 8.31	NA		NA	
Zhang-2 2018^[30]^	China	37	30	44.00 ± 12.24	44.70 ± 10.85	NA		20 (L)/17 (R)	16 (L)/14 (R)	19 (M)/18 (F)	13 (M)/17 (F)	NA		none		96.03 ± 10.59	96.37 ± 11.52	0		0	
Chen 2019^[31]^	China	58	58	NA		4.50 ± 1.10	4.30 ± 1.00	NA		30 (M)/28 (F)	32 (M)/26 (F)	NA		NA		68.80 ± 25.10	68.10 ± 26.50	19	16	11	12
Huang 2019^[32]^	China	31	31	45.98 ± 7.07	43.25 ± 9.86	NA		NA		13 (M)/18 (F)	16 (M)/15 (F)	NA		NA		NA		NA		NA	
Luo 2019^[33]^	China	40	40	65.00 ± 7.00	66.00 ± 7.30	NA		NA		22 (M)/18 (F)	20 (M)/20 (F)	NA		NA		NA		NA		NA	
Zhang 2019^[34]^	China	50	50	55.38 ± 9.18	55.82 ± 9.49	2503.90 ± 861.40	2423.60 ± 817.60	25 (L)/25 (R)	23 (L)/27 (R)	25 (M)/25 (F)	29 (M)/21 (F)	47	46	5	7	NA		NA		NA	
Zhuang 2019^[35]^	China	40	40	45.38 ± 4.65	45.21 ± 4.83	7.12 ± 4.85	7.69 ± 3.12	NA		20 (M)/20 (F)	21 (M)/19 (F)	NA		NA		63.25 ± 2.51	63.33 ± 2.47	0		0	
Jiang 2020^[36]^	China	39	39	46.80 ± 11.50	45.60 ± 11.80	3.20 ± 1.20	3.70 ± 1.80	NA		19 (M)/23 (F)	18 (M)/21 (F)	NA		NA		56.81 ± 12.91	54.95 ± 11.63	0		0	
Li-1 2020^[37]^	China	28	28	42.80 ± 4. 12	43.10 ± 3.65	NA		NA		16 (M)/12 (F)	15 (M)/13 (F)	NA		NA		NA		NA		NA	
Li-2 2020^[38]^	China	128	112	43.50 ± 2.50		NA		137 (L)/103 (R)		65 (M)/63 (F)	52 (M)/60 (F)	105	99	5	7	NA		0		0	
Li-3 2020^[39]^	China	65	65	42.38 ± 7.51	43.46 ± 8.22	8.24 ± 2.81	8.07 ± 2.64	37 (L)/28 (R)	33 (L)/32 (R)	36 (M)/29 (F)	34 (M)/31 (F)	36	35	24	26	68.16 ± 9.88	67.44 ± 10.28	NA		0	
Lu-1 2020^[40]^	China	32	32	49.80 ± 2.50	50.60 ± 2.20	NA		NA		18 (M)/14 (F)	12 (M)/20 (F)	NA		NA		61.74 ± 17.41	61.58 ± 17.63	NA		NA	
Lu-2 2020^[41]^	China	25	25	63.42 ± 7.09	63.28 ± 6.93	3.03 ± 0.68	3.84 ± 0.71	NA		16 (M)/9 (F)	15 (M)/10 (F)	NA		NA		NA		NA		NA	
Yang 2021^[42]^	China	60	60	43.81 ± 3.48	43.27 ± 3.76	5.62 ± 1.12	5.16 ± 1.01	25 (L)/35 (R)	27 (L)/33 (R)	34 (M)/26 (F)	29 (M)/31 (F)	NA		NA		NA		0		0	
Zhang 2021^[43]^	China	40	40	53.90 ± 6.90	53.20 ± 6.10	NA		19 (L)/21 (R)	20 (L)/20 (R)	20 (M)/20 (F)	21 (M)/19 (F)	NA		NA		74.73 ± 21.24	72.90 ± 22.92	NA		NA	
Zhu 2021^[44]^	China	33	33	43.00 ± 12.00	41.00 ± 15.00	5.10 ± 1.40	5.30 ± 2.70	NA		17 (M)/16 (F)	19 (M)/14 (F)	NA		NA		NA		NA		NA	

C = control group, NA = not available, PTA = pure tone average, T = treatment group.

**Table 2:** Details of the treatment conditions.

Study (publication yr)	SSNHL definition	Drug		Mode of administration		Treatment timing		Duration of treatment		Dose and frequency		Foundation treatment	
		T	C	T	C	T	C	T	C	T	C	T	C
Pan 2013-1^[7]^	At least 20 dB hearing loss in 2 CFs occurring within 3 d	MP	P	postaurieal injection	oral	first-line	first-line	10 d	10 d	1 mL/d every 3 d 3 times	50 mg/d for the first 3 d, 10 mg less a d until the 7th d	105 mg ginaton for intravenous infusion, 100 mg vitamin B1 and 0.5 mg mecobalamin administered orally	
Pan 2013-2^[7]^	At least 20 dB hearing loss in 2 CFs occurring within 3 d	MP	MP	postaurieal injection	IV	first-line	first-line	10 d	10 d	1 mL/d every 3 d 3 times	40 mg/d for the first 3 d, 8 mg less a d until the 7th d	105 mg ginaton for intravenous infusion, 100 mg vitamin B1 and 0.5 mg mecobalamin administered orally	
Chen 2014^[8]^	At least 20 dB hearing loss in 2 CFs occurring within 3 d	MP	DEX	postaurieal injection	IV	first-line	first-line	10~14 d	10~14 d	0.5 mL, 40 mg/mL supplemented with 0.2 mL, 2% lidocaine every 3 d	10 mg/d for the first 3 d, 5 mg/d for the following 3 d	70 mg ginaton added with 10 μg alprostadil for intravenous infusion, 0.5 mg mecobalamin via intramuscular injection	
Wang 2014^[9]^	At least 20 dB hearing loss in 2 CFs occurring within 3 d	MP	DEX	postaurieal injection	IV	first-line	first-line	within 15 d	within 15 d	40 mg/d every 3 d until healed or at most 5 times	10 mg/d for the first 3 d, 5 mg/d for the following 4 d	ginaton, mecobalamin and batroxobin administered within 2 wk	
Cao 2015^[10]^	At least 20 dB hearing loss in 2 CFs occurring within 3 d	MP	DEX	postaurieal injection	IV	first-line	first-line	within 15 d	within 15 d	40 mg/d every 3 d until healed or at most 5 times	10 mg/d for the first 3 d, 5 mg/d for the following 4 d	reducing fibrinogen, promoting the microcirculation and neurotrophic drugs administered within 15 d	
Dong 2015-1^[11]^	At least 20 dB hearing loss in 2 CFs occurring within 3 d	MP	DEX	postaurieal injection	IV	first-line	first-line	within 15 d	within 15 d	40 mg supplemented with 0.2 mL lidocaine every 3 d 5 times	10 mg/d for the first 3 d, 5 mg/d for the following 3 d, 2.5 mg/d for the last 3 d	87.5 mg ginaton once per d; 1 mg batroxobin one time on the first d, then 0.5 mg every other d for following d; 500 mg mecobalamin once a d	
Dong 2015-2^[11]^	At least 20 dB hearing loss in 2 CFs occurring within 3 d	MP	DEX	postaurieal injection	IV	first-line	first-line	within 15 d	within 15d	40 mg supplemented with 0.2 mL lidocaine every 3 d 5 times	10 mg/d for the first 3 d, 5 mg/d for the following 3 d, 2.5 mg/d for the last 3 d	87.5 mg ginaton once per d; 1 mg batroxobin one time on the first day, then 0.5 mg every other d for following d; 500 mg mecobalamin once a d	
Qu 2015^[12]^	At least 20 dB hearing loss in 2 CFs occurring within 3 d	MP	MP	postaurieal injection	IV	first-line	first-line	7 d	10 d	40 mg/d every other D for 10 d	85 mg/d for the first 4 d, then 40 mg/d for the following 3 d	blood vessel enlarging, neurotrophic, activating and stasis removing drugs administered for 10 d	
Qin 2015^[13]^	At least 20 dB hearing loss in 2 CFs occurring within 3 d	MP	DEX	postaurieal injection	IV/oral	NA	NA	15 d	15 d	40 mg supplemented with 1 mL lidocaine every 3 d 5 times	10 mg/d intravenous infusion for the first 5 d, 30 mg prednisone taken orally for the following 3 d, 10 mg/d for the other 3 d, 5 mg/d for the last 3 d	25 mg ginaton and neurotrophic drugs administered via intravenous infusion for 15 d	
Wu 2015^[14]^	At least 15 dB hearing loss in 2 CFs occurring within 3 d	MP	P	postaurieal injection	oral	first-line	first-line	14 d	5 d	40 mg/d every 2 d 7 times	1 mg/kg once a d for 3 or 5 d	20 mL ginkgo-damole supplemented with 10 μg alprostadil administered via injection once a d for 14 d	
Cai 2016^[15]^	At least 30 dB hearing loss in 3 CFs occurring within 3 d	MP	DEX/P	postaurieal injection	IV/oral	first-line	first-line	40 d	40 d	40 mg/d every 3 d for 40 d	10 mg/d dexamethasone for the first 5 d, oral prednisone for the following d	25 mg ginaton and neurotrophic drugs administered via intravenous infusion for 40 d	
Fang 2016^[16]^	At least 20 dB hearing loss in 2 CFs occurring within 3 d	MP	P	postaurieal injection	oral	first-line	first-line	5 d	5 d	40 mg/d every other d 3 times	30 mg/d for 5 d	5 mg sibelium, 25000 u vitamin A, 0.1 g vitamin E taken orally every d for 14 d	
Jia 2016^[17]^	At least 20 dB hearing loss in 2 CFs occurring within 3 d	MP	DEX	postaurieal injection	IV	first-line	first-line	5 d	5 d	40 mg/d for 5 d	10 mg/d for 5 d	140 mg ginaton and 10 μg alprostadil supplemented with 0.5 mg cobamamide administered via intravenous infusion once a day for 10 d, 10 μg batroxobin once a d for the first day, then 5 μg/d every other d 3 times	
Li 2016^[18]^	At least 20 dB hearing loss in 2 CFs occurring within 3 d	MP	MP	postaurieal injection	IV	NA	NA	10 d	10 d	40 mg supplemented with 0.5 mL 2% lidocaine once a d for 10 d	40 mg/d for the first 3 d, then half less every 3 d until the 10th d	5 mL ginkgo-damole supplemented with 0.5 mg mecobalamine once a d for 10 d	
Lu 2016^[19]^	At least 20 dB hearing loss in 2 CFs occurring within 3 d	MP	DEX/P	postaurieal injection	IV/oral	NA	NA	15 d	14 d	40 mg supplemented with 1 ml 2% lidocaine every 3 d for 15 d	10 mg/d dexamethasone administered via intravenous infusion for the first 5 d, oral prednisone for the following d	25 mg ginaton administered via intravenous infusion, oral 0.5 mg mecobalamine thrice a d for 15 d	
Zhang 2016^[20]^	At least 20 dB hearing loss in 2 CFs occurring within 3 d	MP	DEX	postaurieal injection	IV	first-line	first-line	5 d	9 d	20 mg/d every other d 3 times	10 mg/d for the first 3 d, 8 mg/d for the following 3 d, 5 mg/d for the last 3 d	vinpocetine administered via intravenous infusion, mecobalamine and ginaton taken orally	
Zhou 2016^[21]^	At least 20 dB hearing loss in 2 CFs occurring within 3 d	DEX	DEX	postaurieal injection	IV	first-line	first-line	10~14 d	10~14 d	0.5 mg supplemented with 0.2 mL lidocaine every 3 d 4 times	10 mg/d for 6 d	alprostadil administered via intravenous infusion, neurotrophic and vitamin drugs taken orally	
Chen 2017^[22]^	At least 20 dB hearing loss in 2 CFs occurring within 2 wk	MP	DEX	postaurieal injection	IV	first-line	first-line	within 15 d	within 15 d	40 mg/d every 2 d 5 times	10 mg/d for the first 3 d, 5 mg/d for the following 4 d	105 mg/d ginaton, 500 μg/d neurotrophic drugs and defibrase administered via intravenous infusion	
Wei 2017^[23]^	At least 20 dB hearing loss in 2 CFs occurring within 3 d	DEX	DEX	postaurieal injection	IV	first-line	first-line	10 d	10 d	5 mg/d every other d for 10 d	5 mg/d every other d for 10 d	mouse nerve growth factor, blood vessel enlarging drugs, vitamins and coenzymes administered via intravenous infusion	
Zhao 2017^[24]^	At least 20 dB hearing loss in 2 CFs occurring within 3 d	DEX	DEX	postaurieal injection	IV	NA	NA	10 d	10 d	5 mg/d every 2 d 5 times	10 mg/d for the first 3 d, 5 mg/d for the following 3 d	blood vessel enlarging, neurotrophic, activating and microcirculation improving drugs for 10 d	
Zhu 2017^[25]^	At least 20 dB hearing loss in 2 CFs occurring within 3 d	MP	MP	postaurieal injection	IV	first-line	first-line	10 d	10 d	40 mg/d for 5 d	40 mg/d for 5 d	105 mg/d ginaton for 10 d, 5~10 BU/d batroxobin every other d 5 times	
Li 2018^[26]^	At least 20 dB hearing loss in 2 CFs occurring within 3 d	DEX	DEX/P	postaurieal injection	IV/oral	NA	NA	10 d	30 d	0.5 mL/d every 2 d for 10 d	10 mg/d dexamethasone for the first 7 d, oral prednisone for the following d	none	
Wang 2018^[27]^	At least 20 dB hearing loss in 2 CFs occurring within 3 d	MP	MP	postaurieal injection	IV	NA	NA	1 0d	10 d	40 mg/d every 2 d 5 times	80 mg/d for the first 4 d, 40 mg/d for the following 3 d	blood vessel enlarging, neurotrophic, activating and stasis removing drugs administered for 10 d	
Zhan 2018^[28]^	At least 20 dB hearing loss in 2 CFs occurring within 3 d	MP	MP	postaurieal injection	IV	NA	NA	NA	NA	10~160 mg per time for 12 times	10~160 mg per time for 12 times	ginaton administered via intravenous infusion	
Zhang-1 2018^[29]^	At least 20 dB hearing loss in 2 CFs occurring within 3 d	MP	DEX/P	postaurieal injection	IV/oral	NA	NA	15 d	15 d	40 mg supplemented with 1 mL 2% lidocaine every 3 d for 15 d	10 mg/d administered via intravenous infusion for a wk, oral prednisone for the following d	none	
Zhang-2 2018^[30]^	At least 20 dB hearing loss in 2 CFs occurring within 3 d	DEX	DEX	postaurieal injection	IV	first-line	first-line	NA	5 d	5 mg per time	10 mg/d administered via intravenous infusion for 5 d	anticoagulation and microcirculation improving drugs administered via intravenous infusion	
Chen 2019^[31]^	At least 20 dB hearing loss in 2 CFs occurring within 3 d	DEX	DEX	postaurieal injection	IV	second-line	second-line	14 d	14 d	40 mg/d every other d 5 times	10 mg/d for the first 3 d, 5 mg/d for the following 2 d	neurotrophic and microcirculation improving drugs administered for 14 d	
Huang 2019^[32]^	At least 20 dB hearing loss in 2 CFs occurring within 3 d	NA	MP	postaurieal injection	IV	NA	NA	6 d	NA	20 mg/d every other day 3 times	0.8 mg/kg·d administered via intravenous infusion	0.5 mg mecobalamine supplemented with 4 mL gastrodin once per d via intravenous infusion	
Luo 2019^[33]^	At least 20 dB hearing loss in 2 CFs occurring within 3 d	MP	MP	postaurieal injection	IV	first-line	first-line	10 d	10 d	0.5 mL/d every other d for 10 d	80 mg/d for the first d, then 20 mg less 3 d until the 10th day	none	
Zhang 2019^[34]^	At least 20 dB hearing loss in 2 CFs occurring within 3 d	MP	MP	postaurieal injection	IV	NA	NA	5 d	10 d	20 mg/d every other d 3 times	0.8 mg/kg·d for the first 5 d, then 8 mg less a d until the 9th d	0.5 mg mecobalamine supplemented with 4 mL gastrodin once per d via intravenous infusion	
Zhuang 2019^[35]^	At least 20 dB hearing loss in 2 CFs occurring within 3 d	MP	DEX/MP	postaurieal injection	IV/oral	first-line	first-line	14 d	14 d	20 mg supplemented with 0.2 mL 2% lidocaine once a d every 3 d 5 times	10 mg/d dexamethasone for the first 5 d, oral prednisone for the following d	10 μg alprostadil supplemented with 0.5 mg mecobalamine once a d for 2 wk	
Jiang 2020^[36]^	At least 20 dB hearing loss in 2 CFs occurring within 3 d	MP	MP	postaurieal injection	IV	first-line	first-line	14 d	14 d	40 mg/d every other d 7 times	80 mg/d for the first 3 d, 40 mg/d for the following 4 d	ginaton, mecobalamine and batroxobin once a d for 2 wk	
Li-1 2020^[37]^	NA	MP	DEX	postaurieal injection	IV	first-line	first-line	15 d	15 d	40 mg/d every other d for 15 d	10 mg/d for the first 3 d, 5 mg/d for the following 4 d	none	
Li-2 2020^[38]^	At least 20 dB hearing loss in 2 CFs occurring within 7 d	MP	DEX	postaurieal injection	IV	NA	NA	10 d	10 d	40 mg/d every other d 5 times	10 mg/d for the first 3 d, 5 mg/d for the following 3 d	105 mg/d ginaton for 10 d	
Li-3 2020^[39]^	At least 20 dB hearing loss in 2 CFs occurring within 7 d	MP	DEX	postaurieal injection	IV	first-line	first-line	10 d	10 d	20 mg/d every other d 5 times	10 mg/d for the first 3 d, 5 mg/d for the following 7 d	ginaton administered via intravenous infusion and oral mecobalamine for 10 d	
Lu-1 2020^[40]^	NA	MP	DEX	postaurieal injection	IV	NA	NA	14 d	6 d	40 mg supplemented with 2% lidocaine every 3 d for 2 wk	10 mg/d for the first 3 d, 6 mg/d for the following 3 d	none	
Lu-2 2020^[41]^	At least 20 dB hearing loss in 2 CFs occurring within 3 d	MP	MP	postaurieal injection	IV	NA	NA	10 d	10 d	40 mg/d every other d for 10 d	40 mg/d for the first 5 d, 8 mg less a day until to the 10th day	neurotrophic and microcirculation improving drugs	
Yang 2021^[42]^	NA	DEX	DEX	postaurieal injection	IV	NA	NA	14 d	14 d	5 mg/d every 3 d	10 mg/d for the first 3 d, 5 mg/d for the following 4 d	20~40 mg/d ginaton and 10 AU/d defibrase supplemented with 0.5 mg/d mecobalamine once a d for 14 d	
Zhang 2021^[43]^	At least 20 dB hearing loss in 2 CFs occurring within 3 d	MP	MP	postaurieal injection	IV	first-line	first-line	10 d	10 d	40 mg/mL once a d for 5 d	40 mg/d for 5 d	30 mL/d ginkgo-damole for 10 d	
Zhu 2021^[44]^	At least 20 dB hearing loss in 2 CFs occurring within 3 d	MP	MP	postaurieal injection	IV	second-line	second-line	15 d	15 d	0.5 mL supplemented with 0.3 mL 2% lidocaine once a d every 3 d 5 times	NA	ginaton administered via intravenous infusion and oral mecobalamine for 2 wk	

C = control group, CF = continuous frequency, d = day, DEX = dexamethasone, M = moth, MP = methylprednisolone, NA = not available, P = prednisone, PTA = pure tone average, SSNHL = sudden sensorineural hearing loss, T = treatment group.

**Table 3:** Summary of the treatment outcomes.

Study (publication yr)	SSNHL definition	Drug		Mode of administration		Treatment timing		Duration of treatment		Dose and frequency		Foundation treatment	
		T	C	T	C	T	C	T	C	T	C	T	C
Pan 2013-1^[7]^	At least 20 dB hearing loss in 2 CFs occurring within 3 d	MP	P	postaurieal injection	oral	first-line	first-line	10 d	10 d	1 mL/d every 3 d 3 times	50 mg/d for the first 3 d, 10 mg less a day until the 7th day	105 mg ginaton for intravenous infusion, 100 mg vitamin B1 and 0.5 mg mecobalamin administered orally	
Pan 2013-2^[7]^	At least 20 dB hearing loss in 2 CFs occurring within 3 d	MP	MP	postaurieal injection	IV	first-line	first-line	10 d	10 d	1 mL/d every 3 d 3 times	40 mg/d for the first 3 d, 8 mg less a day until the 7th day	105 mg ginaton for intravenous infusion, 100 mg vitamin B1 and 0.5 mg mecobalamin administered orally	
Chen 2014^[8]^	At least 20 dB hearing loss in 2 CFs occurring within 3 d	MP	DEX	postaurieal injection	IV	first-line	first-line	10~14 d	10~14 d	0.5 mL, 40 mg/mL supplemented with 0.2 mL, 2% lidocaine every 3 d	10 mg/d for the first 3 d, 5 mg/d for the following 3 d	70 mg ginaton added with 10 μg alprostadil for intravenous infusion, 0.5 mg mecobalamin via intramuscular injection	
Wang 2014^[9]^	At least 20 dB hearing loss in 2 CFs occurring within 3 d	MP	DEX	postaurieal injection	IV	first-line	first-line	within 15 d	within 15 d	40 mg/d every 3 d until healed or at most 5 times	10 mg/d for the first 3 d, 5 mg/d for the following 4 d	ginaton, mecobalamin and batroxobin administered within 2 wk	
Cao 2015^[10]^	At least 20 dB hearing loss in 2 CFs occurring within 3 d	MP	DEX	postaurieal injection	IV	first-line	first-line	within 15 d	within 15 d	40 mg/d every 3 d until healed or at most 5 times	10 mg/d for the first 3 d, 5 mg/d for the following 4 d	reducing fibrinogen, promoting the microcirculation and neurotrophic drugs administered within 15 d	
Dong 2015-1^[11]^	At least 20 dB hearing loss in 2 CFs occurring within 3 d	MP	DEX	postaurieal injection	IV	first-line	first-line	within 15 d	within 15 d	40 mg supplemented with 0.2 mL lidocaine every 3 d 5 times	10 mg/d for the first 3 d, 5 mg/d for the following 3 d, 2.5 mg/d for the last 3 d	87.5 mg ginaton once per day; 1 mg batroxobin one time on the first day, then 0.5 mg every other day for following d; 500 mg mecobalamin once a day	
Dong 2015-2^[11]^	At least 20 dB hearing loss in 2 CFs occurring within 3 d	MP	DEX	postaurieal injection	IV	first-line	first-line	within 15 d	within 15d	40 mg supplemented with 0.2 mL lidocaine every 3 d 5 times	10 mg/d for the first 3 d, 5 mg/d for the following 3 d, 2.5 mg/d for the last 3 d	87.5 mg ginaton once per day; 1 mg batroxobin one time on the first day, then 0.5 mg every other day for following d; 500 mg mecobalamin once a day	
Qu 2015^[12]^	At least 20 dB hearing loss in 2 CFs occurring within 3 d	MP	MP	postaurieal injection	IV	first-line	first-line	7 d	10 d	40 mg/d every other day for 10 d	85 mg/d for the first 4 d, then 40 mg/d for the following 3 d	blood vessel enlarging, neurotrophic, activating and stasis removing drugs administered for 10 d	
Qin 2015^[13]^	At least 20 dB hearing loss in 2 CFs occurring within 3 d	MP	DEX	postaurieal injection	IV/oral	NA	NA	15 d	15 d	40 mg supplemented with 1 mL lidocaine every 3 d 5 times	10 mg/d intravenous infusion for the first 5 d, 30 mg prednisone taken orally for the following 3 d, 10 mg/d for the other 3 d,5 mg/d for the last 3 d	25 mg ginaton and neurotrophic drugs administered via intravenous infusion for 15 d	
Wu 2015^[14]^	At least 15 dB hearing loss in 2 CFs occurring within 3 d	MP	P	postaurieal injection	oral	first-line	first-line	14 d	5 d	40 mg/d every 2 d 7 times	1 mg/kg once a day for 3 or 5 d	20 mL ginkgo-damole supplemented with 10 μg alprostadil administered via injection once a day for 14 d	
Cai 2016^[15]^	At least 30 dB hearing loss in 3 CFs occurring within 3 d	MP	DEX/P	postaurieal injection	IV/oral	first-line	first-line	40 d	40 d	40 mg/d every 3 d for 40 d	10 mg/d dexamethasone for the first 5 d, oral prednisone for the following d	25 mg ginaton and neurotrophic drugs administered via intravenous infusion for 40 d	
Fang 2016^[16]^	At least 20 dB hearing loss in 2 CFs occurring within 3 d	MP	P	postaurieal injection	oral	first-line	first-line	5 d	5 d	40 mg/d every other day 3 times	30 mg/d for 5 d	5 mg sibelium, 25000 u vitamin A, 0.1 g vitamin E taken orally every day for 14 d	
Jia 2016^[17]^	At least 20 dB hearing loss in 2 CFs occurring within 3 d	MP	DEX	postaurieal injection	IV	first-line	first-line	5 d	5 d	40 mg/d for 5 d	10 mg/d for 5 d	140 mg ginaton and 10 μg alprostadil supplemented with 0.5 mg cobamamide administered via intravenous infusion once a day for 10 d, 10 μg batroxobin once a day for the first day, then 5 μg/d every other day 3 times	
Li 2016^[18]^	At least 20 dB hearing loss in 2 CFs occurring within 3 d	MP	MP	postaurieal injection	IV	NA	NA	10 d	10 d	40 mg supplemented with 0.5 mL 2% lidocaine once a day for 10 d	40 mg/d for the first 3 d, then half less every 3 d until the 10th day	5 mL ginkgo-damole supplemented with 0.5 mg mecobalamine once a day for 10 d	
Lu 2016^[19]^	At least 20 dB hearing loss in 2 CFs occurring within 3 d	MP	DEX/P	postaurieal injection	IV/oral	NA	NA	15 d	14 d	40 mg supplemented with 1 mL 2% lidocaine every 3 d for 15 d	10 mg/d dexamethasone administered via intravenous infusion for the first 5 d, oral prednisone for the following d	25 mg ginaton administered via intravenous infusion, oral 0.5 mg mecobalamine thrice a day for 15 d	
Zhang 2016^[20]^	At least 20 dB hearing loss in 2 CFs occurring within 3 d	MP	DEX	postaurieal injection	IV	first-line	first-line	5 d	9 d	20 mg/d every other day 3 times	10 mg/d for the first 3 d, 8 mg/d for the following 3 d, 5 mg/d for the last 3 d	vinpocetine administered via intravenous infusion, mecobalamine and ginaton taken orally	
Zhou 2016^[21]^	At least 20 dB hearing loss in 2 CFs occurring within 3 d	DEX	DEX	postaurieal injection	IV	first-line	first-line	10~14 d	10~14 d	0.5 mg supplemented with 0.2 mL lidocaine every 3 d 4 times	10 mg/d for 6 d	alprostadil administered via intravenous infusion, neurotrophic and vitamin drugs taken orally	
Chen 2017^[22]^	At least 20 dB hearing loss in 2 CFs occurring within 2 wk	MP	DEX	postaurieal injection	IV	first-line	first-line	within 15 d	within 15 d	40 mg/d every 2 d 5 times	10 mg/d for the first 3 d, 5 mg/d for the following 4 d	105 mg/d ginaton, 500 μg/d neurotrophic drugs and defibrase administered via intravenous infusion	
Wei 2017^[23]^	At least 20 dB hearing loss in 2 CFs occurring within 3 d	DEX	DEX	postaurieal injection	IV	first-line	first-line	10 d	10 d	5 mg/d every other day for 10 d	5 mg/d every other day for 10 d	mouse nerve growth factor, blood vessel enlarging drugs, vitamins and coenzymes administered via intravenous infusion	
Zhao 2017^[24]^	At least 20 dB hearing loss in 2 CFs occurring within 3 d	DEX	DEX	postaurieal injection	IV	NA	NA	10 d	10 d	5 mg/d every 2 d 5 times	10 mg/d for the first 3 d, 5 mg/d for the following 3 d	blood vessel enlarging, neurotrophic, activating and microcirculation improving drugs for 10 d	
Zhu 2017^[25]^	At least 20 dB hearing loss in 2 CFs occurring within 3 d	MP	MP	postaurieal injection	IV	first-line	first-line	10 d	10 d	40 mg/d for 5 d	40 mg/d for 5 d	105 mg/d ginaton for 10 d, 5~10 BU/d batroxobin every other day 5 times	
Li 2018^[26]^	At least 20 dB hearing loss in 2 CFs occurring within 3 d	DEX	DEX/P	postaurieal injection	IV/oral	NA	NA	10 d	30 d	0.5 mL/d every 2 d for 10 d	10 mg/d dexamethasone for the first 7 d, oral prednisone for the following d	none	
Wang 2018^[27]^	At least 20 dB hearing loss in 2 CFs occurring within 3 d	MP	MP	postaurieal injection	IV	NA	NA	1 0d	10 d	40 mg/d every 2 d 5 times	80 mg/d for the first 4 d, 40 mg/d for the following 3 d	blood vessel enlarging, neurotrophic, activating and stasis removing drugs administered for 10 d	
Zhan 2018^[28]^	At least 20 dB hearing loss in 2 CFs occurring within 3 d	MP	MP	postaurieal injection	IV	NA	NA	NA	NA	10~160 mg per time for 12 times	10~160 mg per time for 12 times	ginaton administered via intravenous infusion	
Zhang-1 2018^[29]^	At least 20 dB hearing loss in 2 CFs occurring within 3 d	MP	DEX/P	postaurieal injection	IV/oral	NA	NA	15 d	15 d	40 mg supplemented with 1 mL 2% lidocaine every 3 d for 15 d	10 mg/d administered via intravenous infusion for a wk, oral prednisone for the following d	none	
Zhang-2 2018^[30]^	At least 20 dB hearing loss in 2 CFs occurring within 3 d	DEX	DEX	postaurieal injection	IV	first-line	first-line	NA	5 d	5 mg per time	10 mg/d administered via intravenous infusion for 5 d	anticoagulation and microcirculation improving drugs administered via intravenous infusion	
Chen 2019^[31]^	At least 20 dB hearing loss in 2 CFs occurring within 3 d	DEX	DEX	postaurieal injection	IV	second-line	second-line	14 d	14 d	40 mg/d every other day 5 times	10 mg/d for the first 3 d, 5 mg/d for the following 2 d	neurotrophic and microcirculation improving drugs administered for 14 d	
Huang 2019^[32]^	At least 20 dB hearing loss in 2 CFs occurring within 3 d	NA	MP	postaurieal injection	IV	NA	NA	6 d	NA	20 mg/d every other day 3 times	0.8 mg/kg·d administered via intravenous infusion	0.5 mg mecobalamine supplemented with 4 mL gastrodin once per day via intravenous infusion	
Luo 2019^[33]^	At least 20 dB hearing loss in 2 CFs occurring within 3 d	MP	MP	postaurieal injection	IV	first-line	first-line	10 d	10 d	0.5 mL/d every other day for 10 d	80 mg/d for the first day, then 20 mg less 3 d until the 10th day	none	
Zhang 2019^[34]^	At least 20 dB hearing loss in 2 CFs occurring within 3 d	MP	MP	postaurieal injection	IV	NA	NA	5 d	10 d	20 mg/d every other day 3 times	0.8 mg/kg·d for the first 5 d, then 8 mg less a day until the 9th day	0.5 mg mecobalamine supplemented with 4 mL gastrodin once per day via intravenous infusion	
Zhuang 2019^[35]^	At least 20 dB hearing loss in 2 CFs occurring within 3 d	MP	DEX/MP	postaurieal injection	IV/oral	first-line	first-line	14 d	14 d	20 mg supplemented with 0.2 mL 2% lidocaine once a day every 3 d 5 times	10 mg/d dexamethasone for the first 5 d, oral prednisone for the following d	10 μg alprostadil supplemented with 0.5 mg mecobalamine once a day for 2 wk	
Jiang 2020^[36]^	At least 20 dB hearing loss in 2 CFs occurring within 3 d	MP	MP	postaurieal injection	IV	first-line	first-line	14 d	14 d	40 mg/d every other day 7 times	80 mg/d for the first 3 d, 40 mg/d for the following 4 d	ginaton, mecobalamine and batroxobin once a day for 2 wk	
Li-1 2020^[37]^	NA	MP	DEX	postaurieal injection	IV	first-line	first-line	15 d	15 d	40 mg/d every other day for 15 d	10 mg/d for the first 3 d, 5 mg/d for the following 4 d	none	
Li-2 2020^[38]^	At least 20 dB hearing loss in 2 CFs occurring within 7 d	MP	DEX	postaurieal injection	IV	NA	NA	10 d	10 d	40 mg/d every other day 5 times	10 mg/d for the first 3 d, 5 mg/d for the following 3 d	105 mg/d ginaton for 10 d	
Li-3 2020^[39]^	At least 20 dB hearing loss in 2 CFs occurring within 7 d	MP	DEX	postaurieal injection	IV	first-line	first-line	10 d	10 d	20 mg/d every other day 5 times	10 mg/d for the first 3 d, 5 mg/d for the following 7 d	ginaton administered via intravenous infusion and oral mecobalamine for 10 d	
Lu-1 2020^[40]^	NA	MP	DEX	postaurieal injection	IV	NA	NA	14 d	6 d	40 mg supplemented with 2% lidocaine every 3 d for 2 wk	10 mg/d for the first 3 d, 6 mg/d for the following 3 d	none	
Lu-2 2020^[41]^	At least 20 dB hearing loss in 2 CFs occurring within 3 d	MP	MP	postaurieal injection	IV	NA	NA	1 0d	10 d	40 mg/d every other day for 10 d	40 mg/d for the first 5 d, 8 mg less a day until to the 10th day	neurotrophic and microcirculation improving drugs	
Yang 2021^[42]^	NA	DEX	DEX	postaurieal injection	IV	NA	NA	14 d	14 d	5 mg/d every 3 d	10 mg/d for the first 3 d, 5 mg/d for the following 4 d	20~40 mg/d ginaton and 10 AU/d defibrase supplemented with 0.5 mg/d mecobalamine once a day for 14 d	
Zhang 2021^[43]^	At least 20 dB hearing loss in 2 CFs occurring within 3 d	MP	MP	postaurieal injection	IV	first-line	first-line	10 d	10 d	40 mg/mL once a day for 5 d	40 mg/d for 5 d	30 mL/d ginkgo-damole for 10 d	
Zhu 2021^[44]^	At least 20 dB hearing loss in 2 CFs occurring within 3 d	MP	MP	postaurieal injection	IV	second-line	second-line	15 d	15 d	0.5 mL supplemented with 0.3 mL 2% lidocaine once a day every 3 d 5 times	NA	ginaton administered via intravenous infusion and oral mecobalamine for 2 wk	

C = control group, NA = not available, PTA = pure tone average, SSNHL = sudden sensorineural hearing loss, T = treatment.

### 3.2. Risk-of-bias assessment

**Figure 3.**
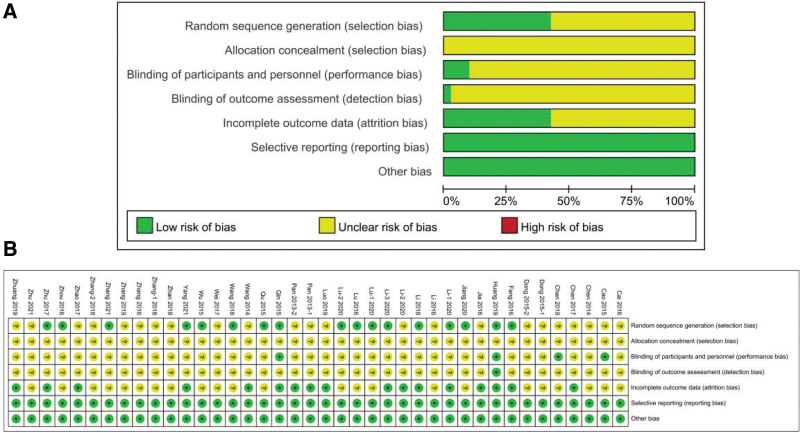
Risk of bias. (A) Risk of bias graph. (B) Risk of bias summary.

Figure 3A and B illustrate the methodological quality of the included studies. All the included studies mentioned random sequence generation. Only 17 studies provided detailed methods of randomization. None of the studies described the concealment of allocation. Regarding the blinding of participants and personnel, only 1 study had an explicit double-blind design,^[32]^ and 3 studies had a single-blind design.^[10,13,31]^ In terms of incomplete outcome data, 16 studies completely described the treatment efficacy and side effect conditions.^[7,9,13,16,17,22,24–26,32,33,35,37–39,42]^ In the domain of selective reporting and other bias, all the studies were judged as having a “low” risk of bias (Fig. 3A and B).

### 3.3. Meta-analysis

#### 3.3.1. RR among patients with SSNHL after steroid treatment.

**Figure 4.**
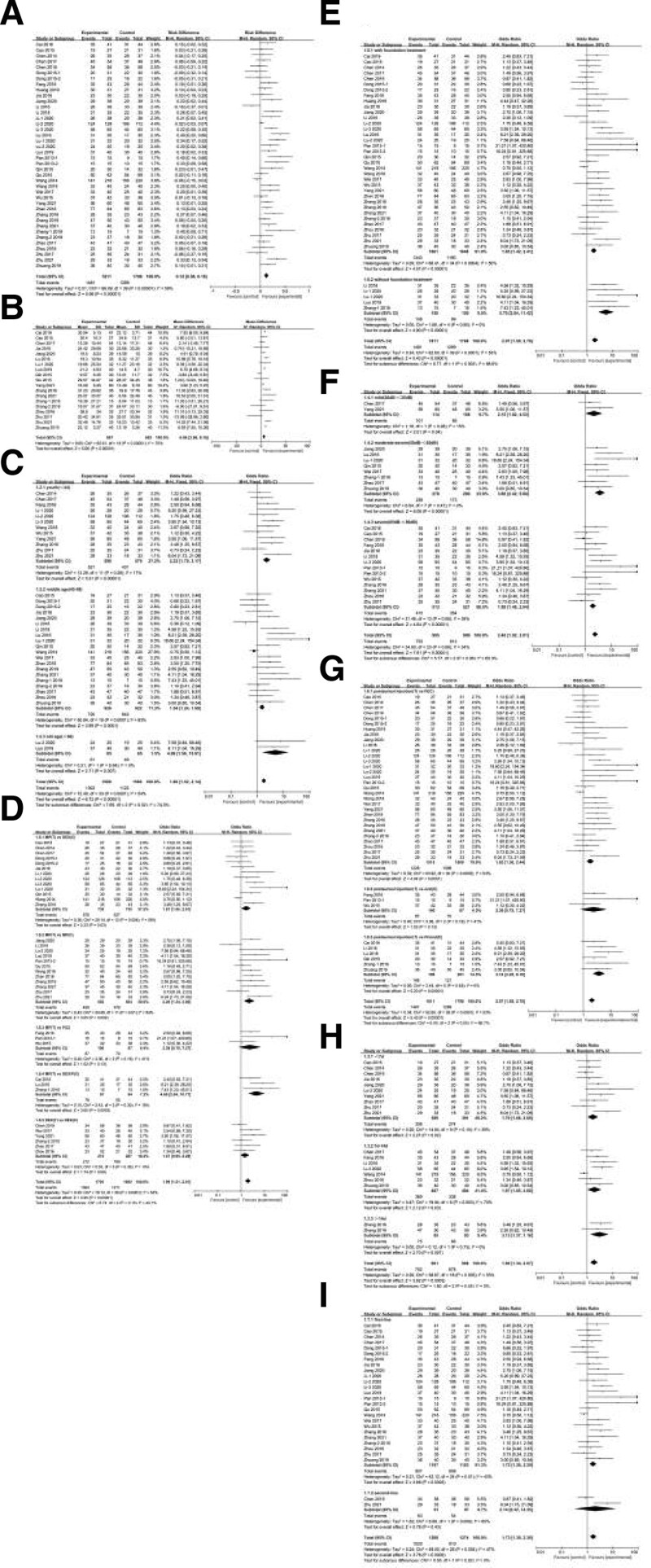
Forest plot. (A) Analysis comparing postauricular injection versus systemic 27 therapy for the recovery rate in SSNHL patients. (B) Analysis comparing postauricular injection versus systemic therapy for variation in the PTA in SSNHL patients. (C–I) Subgroup analysis forest plot. PTA = pure-tone audiometry, SSNHL = sudden sensorineural hearing loss.

All the studies, with a total of 3609 participants, compared the RR of postauricular injection and systemic therapy for SSNHL, which was available for analysis using a random-effects model, with moderate heterogeneity among studies (I^2^ = 59%, *P* < .00001). The results showed statistically significant differences between the postauricular injection group and the systemic therapy group (RD = 0.12; 95% CI = 0.008, 0.16; *P* < .00001), as shown in Figure 4A.

#### 3.3.2. Variation in PTA in patients with sudden hearing loss after steroid treatment.

When comparing the PTA variation level between postauricular injection and systemic therapy in SSNHL, a random effects model was used because of the substantial heterogeneity (I^2^ = 70%, *P* < .00001). The pooled mean difference was 6.06 (95% CI = 3.96, 8.16; *P* < .00001) (Fig. 4B), indicating a statistically significant difference in the change in the PTA level between the 2 groups.

### 3.4. Subgroup analyses

To address the high heterogeneity, we conducted subgroup analyses and categorized patients by age, type of drug, basic treatment, initial PTA, treatment timing, drug administration methods, time of onset, and course of treatment.

#### 3.4.1. Age.

We conducted the first subgroup analysis according to the latest definition for the age groups reported by the WHO (youth, <44; middle age, 45–59; and old age, >60).^[45]^ The heterogeneity decreased in these subgroups, indicating that this factor may have been a source of some of the heterogeneity. The postauricular injection vs systemic therapy hearing recovery RD was 0.12, 0.08, and 0.18 in the youth, middle-age and old-age groups, respectively, which demonstrated that the therapeutic effect of the postauricular injection method may be higher in elderly people (Fig. 4C).

#### 3.4.2. Drug.

As shown in Figure 4D, subgroup analysis was stratified by different types of drugs, that is, methylprednisolone (MP)(T) vs dexamethasone (DEX)(C) or MP(T) vs MP(C) or MP(T) vs P(C) or DEX(T) vs DEX(C). The RD of the therapeutic effect was 0.09 (I^2^ = 66%, 95% CI = 0.02, 0.17, *P* = .01) for MP(T) vs DEX(C), 0.13 (I^2^ = 57%, 95% CI = 0.06, 0.20, *P* = .0006) for MP(T) vs MP(C), 0.18 (I^2^ = 72%, 95% CI = −0.03, 0.38, P0.10) for MP(T) vs P(C), and 0.08 (I^2^ = 0%, RD=, 95% CI = 0.02, 0.15, *P* = .02) for DEX(T) vs DEX(C), which may indicate that MP is more effective when administered via postauricular injection and that different types of drug administration methods may be a source of heterogeneity.

#### 3.4.3. Foundation treatment.

This subgroup was defined based on whether foundational treatment was provided. Thirty-three studies with 3309 participants reported hearing recovery conditions after steroid treatment. In addition, some additional foundation therapies were included in this group (Fig. 4E). The pooled analysis demonstrated a statistically significant difference in the RR in the group that received foundational treatment (I^2^ = 56%, RD = 0.11, 95% CI = 0.06, 0.15). To compare the RR between postauricular injection and systemic therapy without foundational treatment, we included 3 studies with a total of 300 participants. The significant result of this comparison also indicated that postauricular injection may be more effective than systemic therapy (I^2^ = 0%, RD = 0.26, 95% CI = 0.17, 0.34). However, the RD increased in the subgroup without foundation treatment, which demonstrated that additional foundation therapy may not be helpful for postauricular steroid therapy.

#### 3.4.4. Initial PTA.

In terms of the initial PTA, we defined subgroups as having mild (20–35 dB HL), moderate-severe (50–65 dB HL), and severe (65–80 dB HL) hearing loss based on the most updated criteria for hearing levels reported by the WHO.^[46]^ The heterogeneity in these subgroups was markedly reduced, which demonstrated that the initial PTA may be a source of heterogeneity. Two studies were included in the mild hearing loss group (I² = 0%, RD = 0.1, 95% CI = 0.00, 0.20). The RD increased to 0.23 (I^2^ = 46%, 95% CI = 0.16, 0.30, *P* < .00001) for moderate-severe hearing loss and to 0.13 (I^2^ = 44%, 95% CI = 0.08, 0.18, *P* < .00001) for severe hearing loss, which indicated that postauricular injection therapy may be more effective in the severe hearing loss patient group (Fig. 4F).

#### 3.4.5. Mode of application.

In the subgroup analysis of different modes of administration (Fig. 4G), the RD of the RR was 1.10 (I^2^ = 56%, 95% CI = 0.06, 0.14, *P* < .00001) in the postauricular injection group vs the intravenous injection (IV) group, 0.18 (I^2^ = 72%, 95% CI = −0.03, 0.38. *P* = .10) in the postauricular injection group vs the oral (C) group and 0.24 (I² = 15%, 95% CI = 0.15, 0.33, *P* < .00001) in the postauricular injection group vs the IV/C (IV combined with C) group, showing that the steroid therapy effect may not be as good with the oral administration method.

#### 3.4.6. Time to onset.

The studies were divided into 3 groups based on the different times to onset: <7 days, 7 to 14 days, and >14 days. The RD was 0.10 (I^2^ = 35%, 95% CI = 0.03, 0.17, *P* = .007) using the <7 days criterion, 0.12 (I^2^ = 69%, 95% CI = 0.01, 0.22, *P* = .03) in the 7 to 14 days group, and 0.16 (I^2^ = 65%, 95% CI = −0.04, 0.35, *P* = .11) for the > 14 days criterion group (Figure), indicating that postauricular steroid injection may be more effective when the disease is diagnosed and treated in a timely manner (Fig. 4H).

#### 3.4.7. Treatment timing.

Comparing treatment timing showed that the hearing recovery RD was 0.10 (I^2^ = 51%, 95% CI = 0.05, 0.15, *P* < .0001) in the first-line treatment group and 0.15 (I^2^ = 86%, 95% CI = −0.22, 0.51, *P* = .43) in the second-line therapy group. This may demonstrate that the effectiveness of the postauricular injection method may be improved with first-line treatment (Fig. 4I).

### 3.5. Side effects

**Figure 5.**
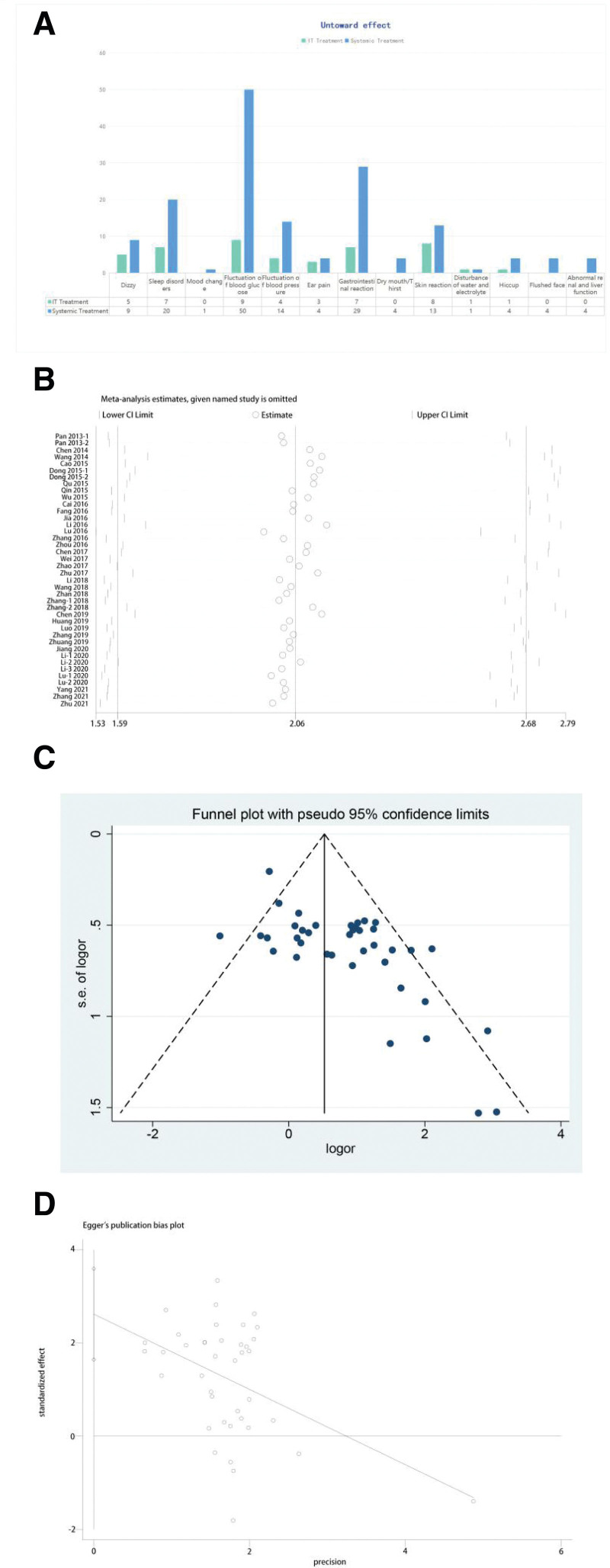
Adverse events and publication bias analysis. (A) Comparison of adverse events between the postauricular injection and systemic therapy groups. (B) Sensitivity analysis of included studies for the recovery rate. (C) Funnel plot of the included studies for the recovery rate. (D) Egger test of the included studies.

Sixteen of the 38 studies reported adverse reactions^[7,9,13,16,17,22,24–26,32,33,35,37–39,42]^ (Fig. 5A). The main adverse effects reported in the systemic therapy group were fluctuations in blood glucose (n = 50), gastrointestinal reactions (n = 29), sleep disorders (n = 20) and fluctuations in blood pressure (n = 14). The postauricular injection group experienced typical side effects of topical treatments, with fluctuations in blood glucose (n = 9) and skin reactions (n = 8) being the most common. Other adverse effects, such as sleep disorders, dizziness and ear pain, were also reported. However, most of these side effects were short-term and minor. In addition, some side effects, such as mood change, dry mouth, and flushed face, were reported only in the systemic group.

### 3.6. Sensitivity analyses

Sensitivity analyses of the RR were performed for the selected studies to identify outliers that affected the overall results (Fig. 5B). We also excluded studies with a high risk of bias, and the results did not change substantially. Therefore, the results of the meta-analysis are reliable.

### 3.7. Publication bias

The potential meta-analysis publication bias was evaluated with funnel plots, as shown in Figure 5C, and the shape of the funnel plots appeared to be approximately symmetric, which indicated no obvious publication bias in our results. Egger test results (P_Egger’s_ = 0.004 > 0) (Fig. 5D) also indicated no significant publication bias among the articles included in the meta-analysis.

## 4. Discussion

Hearing loss can adversely impact patients’ quality of life, and the etiologies and pathophysiological mechanisms have not been completely elucidated.^[2]^ Steroids may represent a widely used and first-line treatment in the clinic.^[47–49]^ However, the treatment efficacy may be largely affected by different administration methods.^[49–51]^ The efficacy of postauricular steroid injection was tested and confirmed by Yang in 2007,^[47]^ who reported that this may be an effective method for patients with contraindications to steroids or persistent sensorineural deafness. Since then, an increasing number of clinicians have started to use postauricular steroid injection as a treatment for SSNHL, and the treatment was also included and recommended in the guidelines for SSNHL in China.^[7–44,52]^ Nevertheless, the efficacy of postauricular injection is still controversial.

In this study, we first developed detailed search strategies and strict inclusion criteria to obtain data for a comprehensive meta-analysis, showing that postauricular injection could effectively improve the hearing levels of SSNHL patients with greater safety. However, the therapeutic effect varies in some ways. The RR is widely used in clinical practice and trials as an outcome assessment. To assess the RR, 38 studies were included, and the pooled result showed that postauricular injection is more likely to provide relief. We also conducted different subgroup analyses based on studies of the RR of SSNHL for several factors.

First, a subgroup analysis by different age groups was performed, with the results suggesting that postauricular injection may be more effective than systemic therapy in all age groups. However, compared with the RD in the young (0.12) and middle-aged groups (0.08), the RD in the old-aged group was increased to 0.18, which indicated that postauricular injection may be more effective for elderly patients. However, the result should be interpreted with caution due to the higher heterogeneity in the youth and middle-age groups. A second subgroup analysis was conducted according to the different types of drugs, and the results demonstrated that MP may increase the therapeutic efficacy of postauricular injection; at the same time, DEX is more suitable for use in systemic therapy. Third, in view of most of the original studies that included some foundational treatment, such as vasodilators and vasoactive therapy, we defined subgroups according to whether any additional therapy was included in the treatment strategy. The results illustrated that foundational therapy may not be helpful with postauricular injection. However, this kind of additional treatment could be combined with systemic steroid therapy to achieve higher efficacy. Fourth, the initial PTA was analyzed based on different levels, and the results indicated that the treatment efficacy of postauricular injection was higher in the moderate-severe and severe hearing loss groups. The baseline data should be improved, including the initial PTA level and types of auditory curves, and different types of SSNHL should be classified in detail for personalized and precise therapy in the future. Fifth, the control group was categorized by 3 different modes of administration, including IV, oral and IV combined with oral steroid therapy. The results demonstrated that the combination of different methods of systemic therapy may lead to higher efficacy. Sixth, in terms of different time durations before treatment, we analyzed 3 subgroups, and the results indicated that prompt treatment is necessary for postauricular injection efficacy in SSNHL. Finally, treatment efficacy was analyzed based on treatment timing, and the results showed that the therapeutic efficacy of postauricular injection may be more suitable for first-line therapy in SSNHL.

### 4.1. Conditions of relevant existing studies

Numerous systematic reviews and meta-analyses have been conducted to compare the therapeutic efficacy of postauricular injection and systemic therapy. The first study was conducted by Wang^[48]^ in 2018, who included a total of 1913 cases, and the results showed evident differences in the total effectiveness rate and cure rate between systemic and postauricular steroid injection treatments (RR = 1.12, 95% CI = 1.05–1.09, *P* = .0003; RR = 1.25, 95% CI = 1.10–1.41, *P* = .0004); the study also observed fewer complications associated with postauricular injection than with systemic therapy. Since then, 3 related meta-analyses^[49–51]^ have been conducted successively, and the results were the same. Liu concluded that the curative effect of the retroauricular injection group was better than that of the systemic hormone group, with an RR (95% CI) of 1.29 (1.19, 1.40). Jiang reported that the postauricular injection group had better outcomes than the systemic therapy group in the treatment of sudden deafness (OR = 3.18, 95% CI = 2.13–4.76, *P* < .00001). Finally, in the study conducted by Mao, the results of the meta-analysis showed that the total clinical efficacy of postauricular injection was better than that of systemic treatment in the low-medium frequency subgroup (*P* < .05).

### 4.2. Strengths and weaknesses of the study

First, to our knowledge, this is the first meta-analysis that included RCTs in English and Chinese literature and analyzed subgroup variables such as age, drug, foundational treatment, initial PTA, mode of administration, time to onset and treatment timing. Second, to ensure the reliability of the conclusions, the literature search was comprehensive, encompassing 8 large databases. Furthermore, the systematic review was performed in accordance with the Preferred Reporting Items for Systematic Review and Meta-analysis Protocols guidelines, including a systematic evaluation of quality and treatment outcomes of all studies published to date and ultimately including high-quality RCTs related to this topic. Finally, sensitivity analyses in this meta-analysis confirmed the validity of our conclusion, and thus, the rationality and reliability of our meta-analysis results was apparently improved. Given the emergence of new evidence regarding postauricular steroid injection for SSNHL, we believe that this route is more effective and safer than systemic therapy.

However, the following limitations of this study need to be considered. First, the original studies included in this meta-analysis exhibited a risk of bias. Most articles did not explain their specific methods of randomization and concealment. Second, different factors, such as age, types of drug, and baseline data, may have led to some heterogeneity in the results. To address this, we defined subgroups, and the heterogeneity was reduced slightly. However, some other probable contributors, such as sex, affected ear, and diabetic status, were not analyzed because of the small sample size and lack of detailed classification in the original studies. Third, some studies included nonsteroid therapy, such as vasoactive and vasodilator drugs, as part of the treatment. These substances may potentially affect the outcomes or be a source of heterogeneity. However, we conducted subgroup analyses according to this factor, and the heterogeneity was reduced. Fourth, few high-quality studies on postauricular injection treatment for patients with diabetes or hypertension are available, making it impossible to analyze these subgroup variables. When discussing treatment safety, it is essential to classify and include this kind of patient. Fifth, all the included studies were published in Chinese, and the participants were Chinese, which may contribute to some risk of bias. Finally, in terms of the side effects, only 42% of the included studies provided the relevant data, and most of them did not describe the detailed condition or prognosis of the side effect in the long term, especially among patients with underlying diseases.

### 4.3. Future practice and research

From our study, we suggest that future studies should focus on the following: Categorizing and grouping SSNHL participants according to detailed baseline data. Administering treatment to elderly individuals and observing and evaluating patients with some underlying conditions, such as diabetes, cerebrospinal-cardiovascular disease, hypertension and epilepsy, with long-term follow-up to elucidate the safety profile and wide applicability of the therapy. Using postauricular injection in a wide range of applications in different countries. Using some new techniques, including microneedle transdermal delivery and sustained-release materials that enable direct steroid delivery to the inner ear for a longer duration of therapeutic drug application. These combination of these techniques with postauricular injection should be promising and helpful for achieving higher therapeutic efficacy and noninvasive effects. Addressing the adverse reactions reported by some studies in recipients of postauricular injections, including fluctuations in blood glucose, skin reactions, sleep disorders, dizziness and ear pain. How to resolve or reduce these adverse reactions is a topic worthy of study (e.g., whether co-injection with lidocaine would reduce ear pain without diminishing efficacy).

## 5. Conclusion

In summary, this is the latest and largest sample meta-analysis comparing postauricular injection and systemic steroid administration routes in SSNHL treatment, involving 3609 participants, and postauricular injection showed better efficacy than systemic therapy, regardless of the age, drug used, basic treatment, initial PTA, drug administration method, time of onset, and course of treatment. Nonetheless, although postauricular injection is a promising therapy for SSNHL, our conclusions are based on a relatively small number of trials and should thus be interpreted with caution. Further prospective, randomized and multicenter studies are needed to confirm these findings.

## Acknowledgments

We acknowledge all the contributing authors.

## Author contributions

**Data curation:** Y. W. Hou.

**Formal analysis:** J. N. Zhang.

**Methodology:** H. S. Deng.

**Writing – review & editing:** T. Yang.
